# Identification and validation of a robust autophagy-related molecular model for predicting the prognosis of breast cancer patients

**DOI:** 10.18632/aging.203187

**Published:** 2021-06-29

**Authors:** Jian-Ying Ma, Qin Liu, Gang Liu, Shasha Peng, Gaosong Wu

**Affiliations:** 1 Department of Thyroid and Breast Surgery, Zhongnan Hospital of Wuhan University, Wuhan, China; 2 Department of Breast Surgery, Thyroid Surgery, Huangshi Central Hospital of Edong Healthcare Group, Hubei Polytechnic University, Huangshi, Hubei, China; 3 Department of Hepatobiliary Surgery, Huangshi Central Hospital of Edong Healthcare Group, Hubei Polytechnic University, Huangshi, Hubei, China

**Keywords:** breast cancer, autophagy, risk, GEO

## Abstract

Despite a relatively low mortality rate, high recurrence rates represent a significant problem for breast cancer (BC) patients. Autophagy affects the development, progression, and prognosis of various cancers, including BC. The aim of the present study was to identify candidate autophagy-related genes (ARGs) and construct a molecular-clinicopathological signature to predict recurrence risk in BC. A 10-ARG-based signature was established in a training cohort (GEO-BC dataset GSE25066) with LASSO Cox regression and assessed in an independent validation cohort (GEO-BC GSE22219). Significant differences in recurrence-free survival were observed for high- and low-risk patients segregated based on their signature-based risk score. Time-dependent receiver operating characteristic (tdROC) analysis of signature performance demonstrated satisfactory accuracy and predictive power in both the training and validation cohorts. Moreover, we developed a nomogram to predict 3- and 5-year recurrence-free survival by combining the autophagy-related risk score and clinicopathological data. Both the tdROC and calibration curves indicated high discriminating ability for the nomogram. This study indicates that our ARG-based signature is an independent prognostic classifier for recurrence-free survival in BC. In addition, individualized survival risk assessment and treatment decisions might be effectively improved by implementing the proposed nomogram.

## INTRODUCTION

Breast cancer (BC) is the most common malignancy in female patients worldwide. According to the latest report of the World Health Organization’s International Agency for Research on Cancer, approximately 2.7 million women were diagnosed with BC and approximately 42, 170 patients died from the disease in the United States in 2020 [1, 2]. Although the overall survival rate has greatly improved over the past decades, most of BC-related deaths are still caused by tumor relapse with or without metastatic progression [3]. BC is a heterogeneous disease, and routine diagnosis and treatment often fail to achieve good effect in some patients. Hence, novel and more reliable molecular biomarkers for diagnosis, treatment, and, prediction of the prognosis of patients with BC are urgently required.

Autophagy is an intracellular evolutionarily conserved catabolic degradation process that maintains cellular homeostasis by degrading senescent organelles and proteins. Studies have shown that autophagy affects the development of multiple cancers, either by recycling biosynthetic components to stimulate tumor growth (especially at advanced stages), or by triggering apoptosis to destroy cancerous or pre-cancerous cells [4–6]. Notably, autophagy can also affect the relationship between normal and tumor cells by alleviating cellular stress and suppressing antitumor immune responses [7]. Hence, in many cancers autophagy is closely correlated with drug resistance, tumor metastasis, and patient prognosis [8, 9].

Postoperative prognosis is traditionally based on the tumor-node-metastasis (TNM) staging system, a paradigm based on tumor size, depth of invasion, number of metastatic lymph nodes, and presence of distant metastasis. This system represents an excellent common language in the field of BC, but excludes prognostic factors such as age, Ki67 status, or expression of tumor-specific molecular markers. Therefore, comprehensive risk-stratified tools involving treatment selection and demographic factors should be created for BC. There is no information on the prognostic value of autophagy-related genes (ARGs) in patients with BC. Therefore, we used bioinformatics to identify candidate ARGs and construct a novel molecular-clinicopathological signature for BC recurrence.

## RESULTS

### Development and validation of an autophagy-related prognostic gene signature for breast cancer

**Figure 1:**
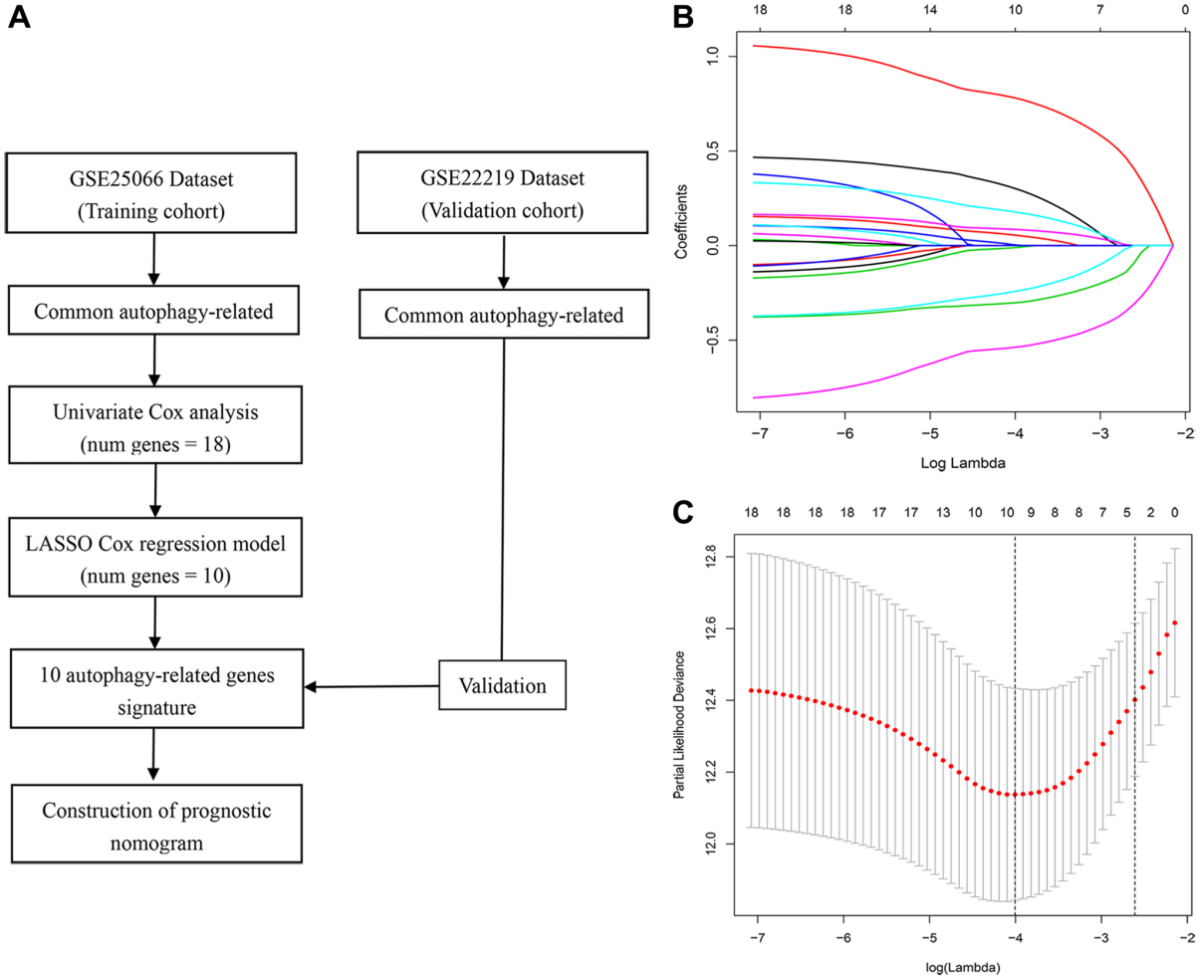
**Study workflow and parameter selection.** (**A**) Workflow of the construction and validation of the signature. (**B**) Ten-time cross-validation for tuning parameter selection in the LASSO Cox regression model. (**C**) Coefficient profiles of 18 autophagy genes.

The study design is illustrated in Figure 1A. After the initial selection process, expression data from 303 and 216 breast cancer patients (reported in the GSE25066 and GSE22219 datasets, respectively) were used as the training and validation cohorts, respectively. After matching the mRNA expression data from the Human Autophagy Database’s autophagy gene list, a total of 219 autophagy-related genes (ARGs) were identified in the training cohort. Baseline clinical features of the studied cases are shown in Table 1.

**Table 1:** Baseline characteristics of study patients.

**Variables**	**Training cohort**	**Validation cohort**
	**No. (%)**	**No. (%)**
No. of patients	303	216
Age (years)	49 (26–75)	55 (26–80)
ER status		
Negative	131 (43.2)	/
Positive	172 (56.8)	/
PR status		
Negative	162 (53.5)	82 (38.0)
Positive	141 (46.5)	134 (62.0)
HER2 status		
Negative	389 (95.4)	/
Positive	3 (1.00)	/
Unknown	11 (3.6)	/
Grade		
I	19 (6.3)	41 (19.0)
II	114 (37.6)	87 (40.3)
III	149 (49.2)	63 (29.2)
Unknown	21 (6.9)	25 (11.5)
T stage		
T1	22 (7.3)	/
T2	162 (53.5)	/
T3	71 (23.4)	/
T4	48 (15.8)	/
N stage		
N0	86 (28.4)	/
N1	149 (49.1)	/
N2	38 (12.5)	/
N3	30 (1.0)	/
Stage		
I	9 (3.0)	/
II	163 (53.8)	/
III	131 (43.2)	/

**Figure 2:**
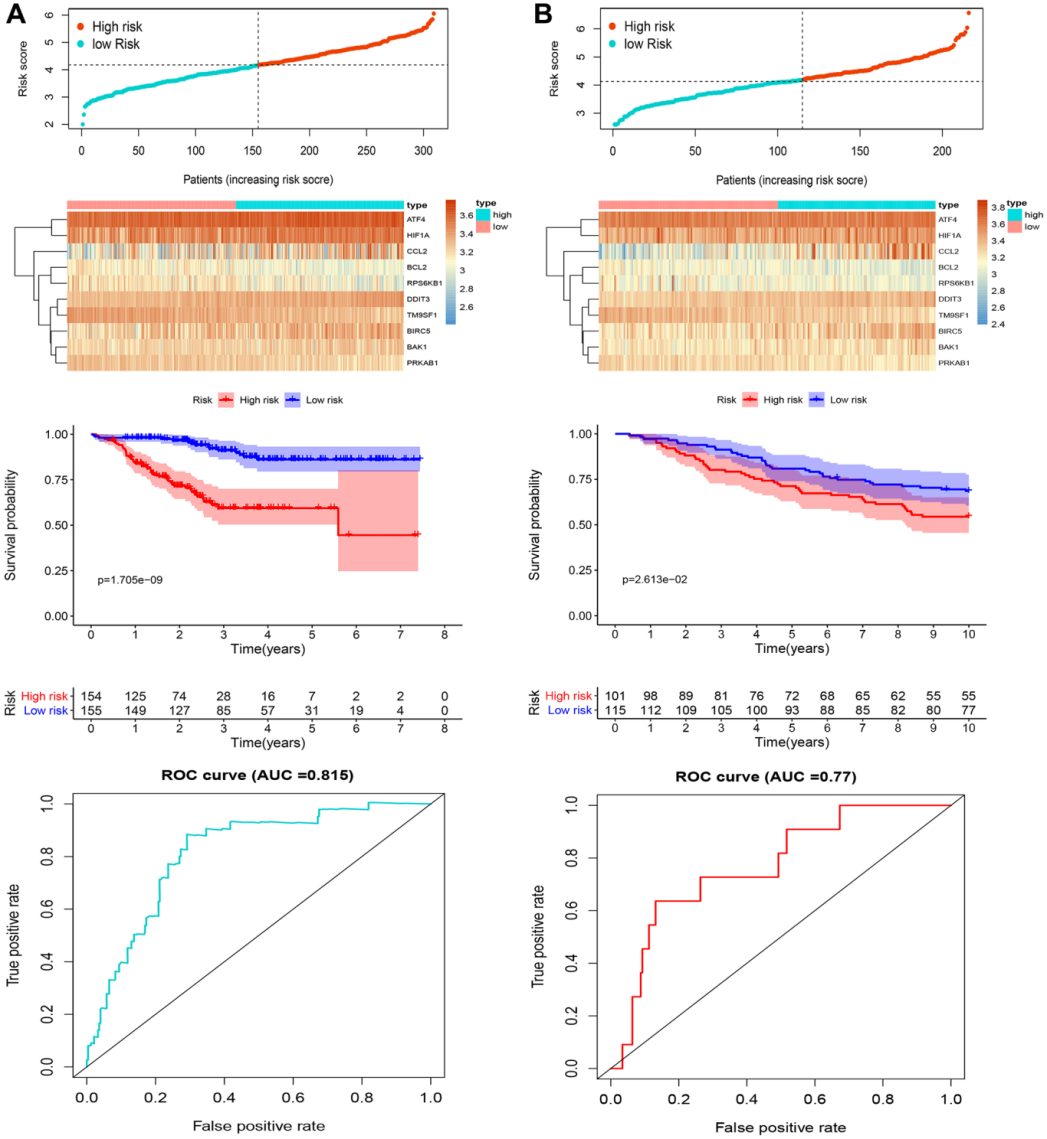
**Analysis of candidate ARGs.** Distribution of risk score, heatmap representation, Kaplan-Meier survival curves, and ROC curves for the autophagy-related signature in (**A**) the training cohort and (**B**) the validation cohort.

**Figure 3:**
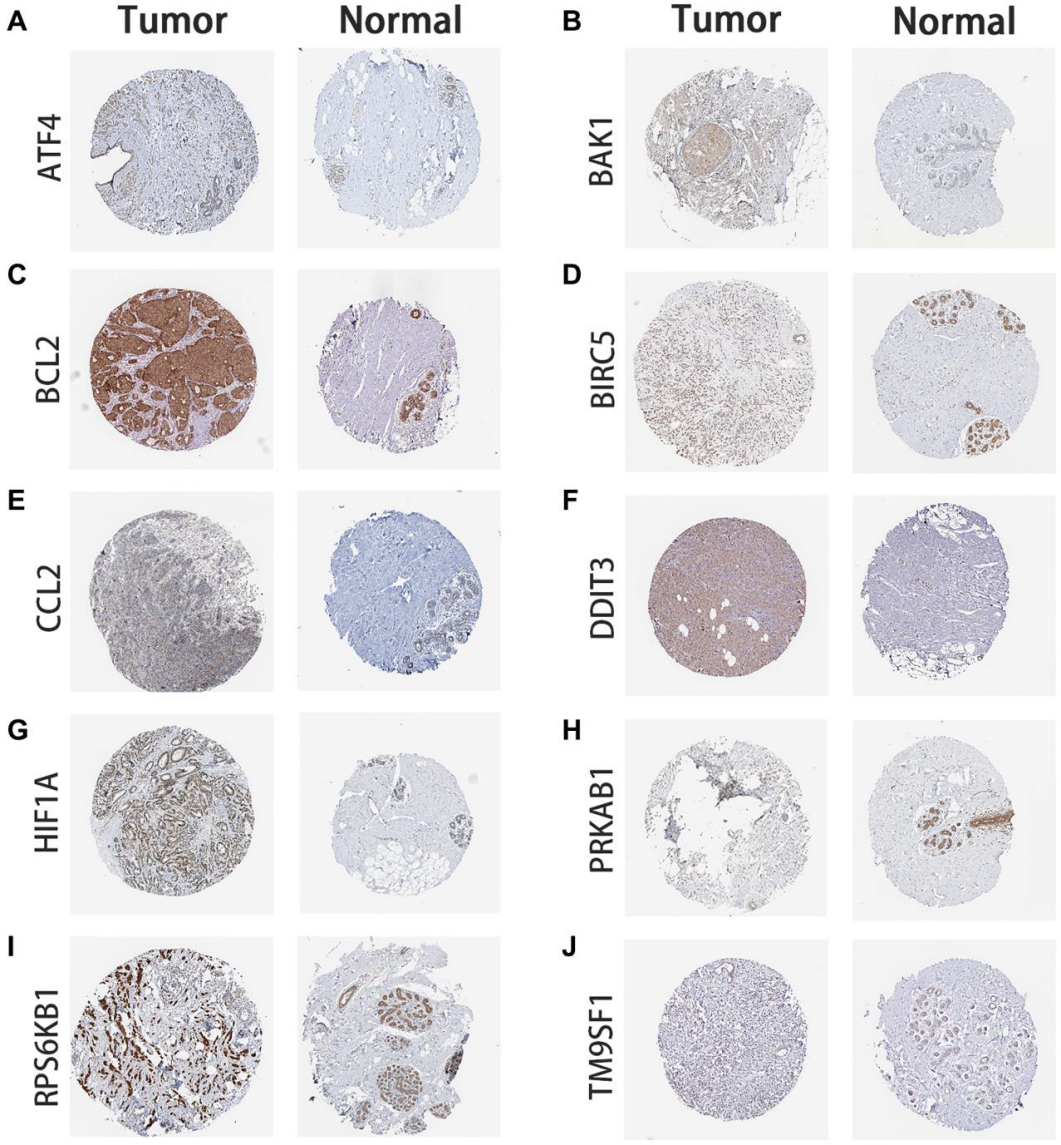
**Immunohistochemistry of ARG expression.** BC tumor and normal breast tissue images are shown for the signature’s ARG-coded proteins. (**A**) ATF4 expression. (**B**) BAK1 expression. (**C**) BCL2 expression. (**D**) BIRC5 expression. (**E**) CCL2 expression. (**F**) DDIT3 expression. (**G**) HIF1A expression. (**H**) PRKAB1 expression. (**I**) RPS6KB1 expression. (**J**) TM9SF1 expression. Images were obtained from the Human Protein Atlas database (https://www.proteinatlas.org/).

To identify genes related to recurrence-free survival (RFS), univariate Cox analysis was performed on 145 ARGs in the training cohort. We found that 18 ARGs were correlated with RFS (*P* < 0.05). Then, we performed LASSO Cox regression analysis and identified, among those, 10 recurrence-related genes (ATF4, BAK1, BCL2, BIRC5, CCL2, DDIT3, HIF1A, PRKAB1, RPS6KB1, and TM9SF1). These ARGs were then used to establish a prognostic signature (Figure 1B and 1C). The risk score was defined by computing the products of the mean LASSO β-coefficients by the corresponding expression level for each gene: (0.31532 × expression of ATF4) + (0.05986 × expression of BAK1) + (–0.013523 × expression of BCL2) + (0.183910 × expression of BIRC5) + (0.08745 × expression of CCL2) + (0.789860 × expression of DDIT3) + (0.008910 × expression of HIF1A) + (–0.305302 × expression of PRKAB1) + (0.24959 × expression of RPS6KB1) + (–0.54193 × expression of TM9SF1). We then divided patients into high- and low-score sub-populations and found that the ARG score was negatively related to prognosis in the training cohort (Figure 2). The signature’s risk score showed excellent ability (AUC = 0.815) in predicting breast cancer patients’ survival risk (Figure 2A). To validate the accuracy of the model in predicting OS, individual risk scores were calculated in patients from an independent dataset (GSE2219; validation cohort). This analysis demonstrated that patients in the high-risk group had worse RFS (*p* < 0.001; AUC = 0.77; Figure 2B). Thus, concordant results were obtained in the training and validation cohorts. Moreover, immunohistochemistry data from the Human Protein Atlas largely confirmed that the signature’s ARGs were dysregulated in BC samples. Specifically, expression levels of ATF4, BAK1, BCL2, CCL2, DDIT3, HIF1A, and RPS6KB1 were higher in BC than in normal breast tissue, whereas the opposite pattern was found for PRKAB1. In contrast, the expression of BIRC5 and TM9SF1 in tumor and normal tissues did not differ (Figure 3).

### Comparison with other prognostic signatures

A comparison of our signature with 6 previously published BC prognostic models was next performed (Table 2). Time-dependent ROC curve analysis of our signature’s ability to predict 3- and 5-years RFS yielded AUC values of 0.815 and 0.765, respectively. These values were comparable to those reported for 9-TF [10] and 12-lncRNA [11] signatures, and superior in turn to the AUC values of another four signatures [12–15].

**Table 2:** The AUC of ROC curve show the sensitivity and specificity of the known signatures in predicting the prognosis of BC patients.

**Author**	**Year**	**Gene signature**	**AUC for RFS**
Tang et al.	2019	13-miRNA signature	0.676 (5-year)
Chen et al.	2020	9-TF signature	0.794 (1-year), 0.822 (3-year), 0.843 (5-year)
Zhang et al.	2020	10-lncRNA signature	0.741 (1-year), 0.752 (3-year), 0.781 (5-year)
Feng et al.	2021	5-gene metabolic signature	0.769 (3-year)
Zhou et al.	2016	12-lncRNA signature	0.847 (5-year)
Lai et al.	2019	5-miRNA signature	0.710 (5-year)

### Correlation of the ARG signature with clinicopathological characteristics

**Figure 4:**
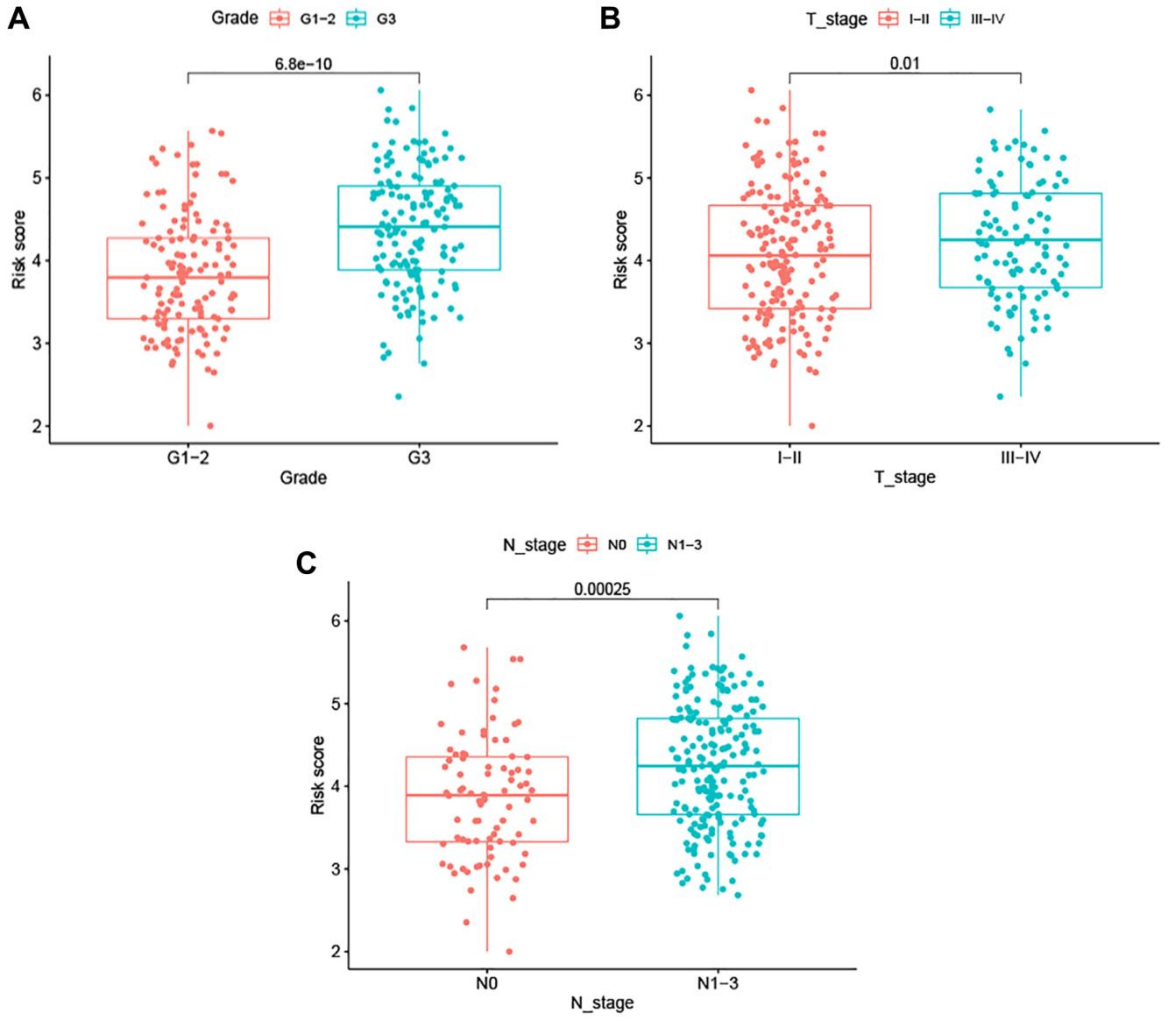
**Association between the ARG-based signature and clinicopathological characteristics.** (**A**) Tumor grade. (**B**) T stage. (**C**) N stage.

Correlation analysis between the signature risk score and seven clinicopathological factors revealed that the signature was not associated with age, but was instead significantly correlated with tumor grade, as well as T and N stage, in BC patients (Figure 4). These data suggest that our ARG signature may reliably predict tumor progression in BC patients.

### Construction of a predictive nomogram for BC

**Figure 5:**
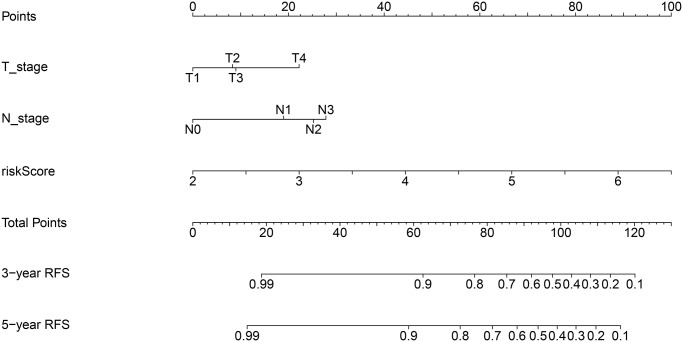
**Nomogram for predicting 3- and 5-year RFS of BC patients.** The nomogram was constructed by integrating ARG signature’s risk score and patient’s T and N stage data.

**Figure 6:**
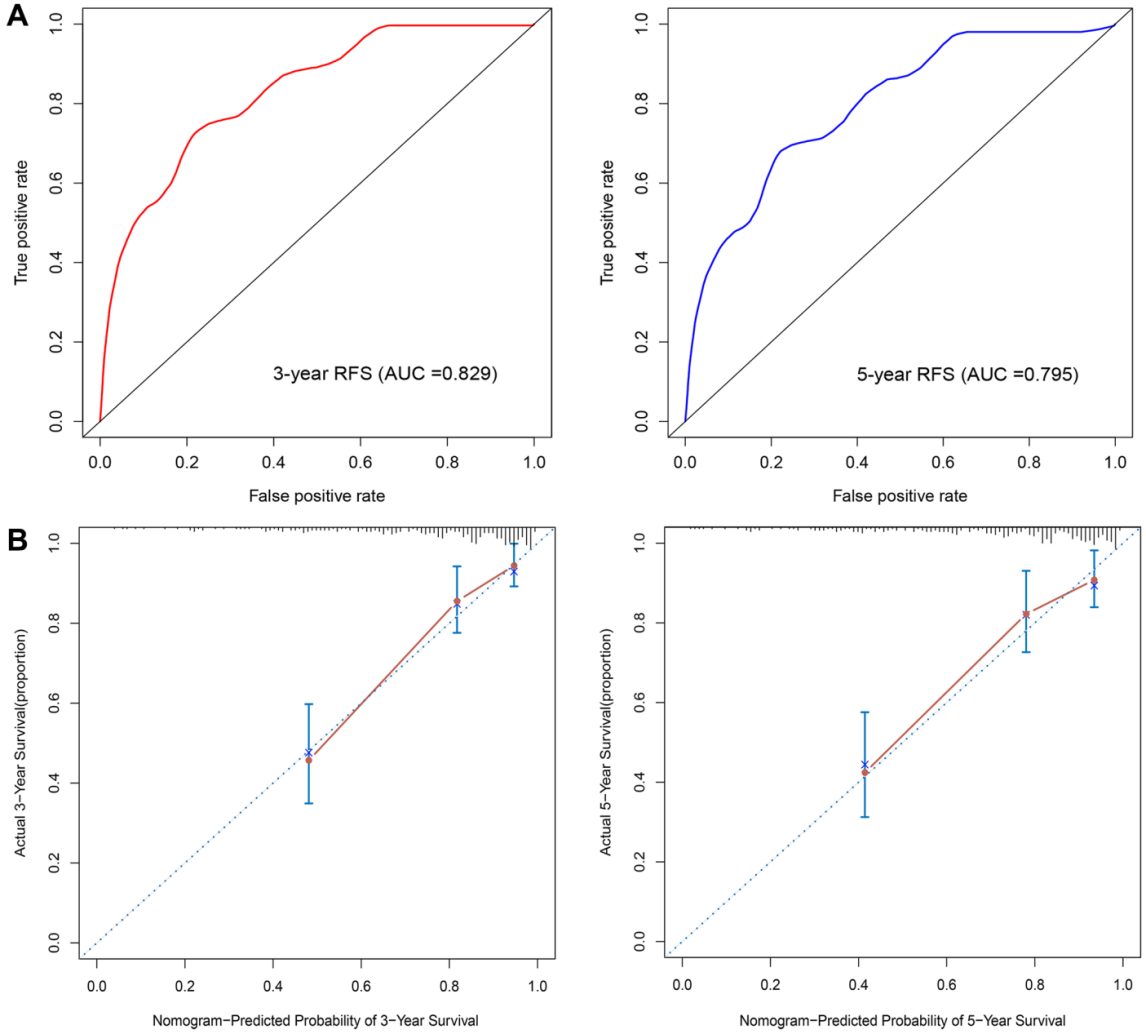
**Nomogram validation.** (**A**) Time-dependent ROC analysis. (**B**) Calibration curves for predicting 3- and 5-year RFS in BC patients.

Univariable Cox regression analyses revealed that risk score, grade, ER status, PR status, and T and N stage were significantly associated with RFS. These 6 variables were next subjected to multivariate Cox regression analysis, from which we constructed a nomogram to predict the RFS of patients by integrating T and N stage data with the risk score of the prognostic signature (Figure 5). By combining the scores associated with each variable and projecting the total score to the bottom scale, the estimated 3-year and 5-year RFS probabilities can be easily calculated. The 3-year and 5-year AUC values of the nomogram were 0.829 and 0.795, respectively (Figure 6A). The ROC curves also indicated that compared with the signature only, the nomogram combining the signature and clinical variables had greater predictive accuracy. Calibration curves were then generated to graphically demonstrate the consistency between nomogram prediction and actual prognosis (Figure 6B).

## DISCUSSION

Despite breakthrough advancements in BC treatment, some BC patients still have a poor prognosis, especially when metastasis is detected. Autophagy plays different roles in different stages of tumorigenesis and in response to anti-tumor treatments. Studies have shown that autophagy is induced by almost all conventional BC treatments and is thus considered a target for clinical pharmacological blockade [16–18]. BC is a multifactorial disease that involves the participation of numerous dysregulated ARGs in tumorigenesis and progression. Therefore, a signature model that capitalizes this important information might provide a more accurate and detailed diagnosis and prognosis prediction than single gene-based predictive models.

In the present study, a total of 219 ARGs were identified in BC samples. Based on LASSO Cox analysis, we then established a 10-ARG-based signature to predict BC recurrence and observed significant differences in RFS for high- and low-risk score patients. The flexibility of LASSO Cox regression analysis allows to perform dimensional analyses more effectively, to construct more accurate genetic disease models and to improve the predictive ability of the corresponding molecular signatures [19]. We validated the predictive accuracy of the model in two independent GEO sets and confirmed the reliability of the model in the GSE22219 dataset. Then, a nomogram integrating risk score, T stage, and N stage was established in the training cohort. ROC curves and calibration plots showed excellent predictive ability for the model. By providing a visual, easily interpretable method for predicting individual RFS in BC patients, our novel nomogram may represent a valuable tool to guide individualized BC therapy.

Our BC signature includes 10 autophagy-associated, recurrence-related genes (ATF4, BAK1, BCL2, BIRC5, CCL2, DDIT3, HIF1A, PRKAB1, RPS6KB1, and TM9SF1). Among these, several have been previously investigated in BC. ATF4, a basic region-leucine zipper transcription factor, belongs to the ATF/CREB (activating transcription factor/cyclic AMP response element binding protein) family [20]. ATF4 overexpression was found to be associated with tumorigenesis in a variety of cancers, including BC [20–22]. During the integrated stress response (ISR), ATF4 regulates tumor growth, autophagy, drug resistance, and metastasis through the PERK and GCN2 pathways [23–25]. Milani et al. found that resistance of BC cells to the 26S proteasome inhibitor bortezomib relies on proteasomal stabilization of ATF4, which upregulates LC3B to activate autophagy [26]. Similarly, apoptosis inhibition in paclitaxel-treated BC cells was also shown to result from ATF4-dependent autophagy activation [27]. These results suggest that ATF4 might be a reliable biomarker for poor prognosis in BC and that targeting ATF4-induced autophagy may overcome BC resistance to various chemotherapies. Gao et al. found that ATF4 expression is upregulated in ER-negative BC and its expression is positively correlated with that of PSAT1, an enzyme involved in the serine synthesis pathway. Through *in vitro* and *in vivo* experiments, they showed that ATF4 silencing can reduce PSAT1 expression and inhibit cell proliferation and tumorigenesis by blocking GSK3β/β-catenin/cyclin D1 signaling [22]. In turn, Zeng et al. revealed that ATF4 is overexpressed in HER2-positive BC, where it upregulates ZEB1 and inhibits E-cadherin expression to promote cell migration [28].

C-C motif chemokine 2 (CCL2, also known as MCP-1) belongs to the CC chemokine family that recruits monocytes, memory T cells, and dendritic cells to sites of inflammation [29, 30]. Studies have shown that CCL2 in the tumor microenvironment promotes the progression and metastasis of different tumors, including BC [31, 32]. Indeed, CCL2 expression is often increased in BC tissues, and high CCL2 expression is associated with early recurrence and worse prognosis in BC [33, 34]. Fang et al. reported that CCL2 is significantly overexpressed in human luminal B BC specimens, as well as in MMTV-PyVmT and MMTV-Neu transgenic mammary tumors. Overexpression of CCL2 in luminal B cancer cells promoted cell growth and survival by inhibiting necrosis and autophagy [35]. DNA damage-inducible transcript 3 (DDIT3, also known as GADD153, or CHOP) is a member of the CCAAT/enhancer-binding proteins (C/EBPs). DDIT3 is regulated by ATF4 and acts as a multifunctional transcription factor during the ER-stress response [36, 37]. Block et al. indicated that properdin inhibited BC cell growth through testin-mediated DDIT3 upregulation [38]. In turn, Tan et al. revealed that DDIT3 was significantly up-regulated in T-47D breast cancer cells, and its silencing inhibited the formation of ER vacuoles and autophagosomes [39].

In conclusion, we established an ARG signature that can accurately predict RFS in BC patients. In addition, our nomogram combining risk score and clinical parameters can provide visual individualized estimates of potential survival benefits, which may aid the design of patient-tailored therapies. There are some limitations in the current research. Our signature was established using a computational frame, and although our quantitative prognostic model proved to be robust, the mechanisms by which some of the signature genes may modulate BC progression have not yet been elucidated. Therefore, *in vitro* and *in vivo* functional experiments are required to verify their biological effects.

## MATERIALS AND METHODS

### Dataset acquisition and pre-processing

The GSE25066 and GSE22219 gene expression profiles were acquired from the GEO database (https://www.ncbi.nlm.nih.gov/geo/). The GSE25066 dataset is based on the GPL96 [HG-U133A] Affymetrix Human Genome U133A Array and includes data from 310 breast cancer patients. The GSE22219 microarray dataset is based on the GPL6098 Illumina human Ref-8 v1.0 expression beadchip and contains data from 216 breast cancer patients. The GSE25066 dataset was used as training set and the GSE22219 dataset was used as validation set. The autophagy gene list was obtained from the Human Autophagy Database (HADb, http://autophagy.lu/clustering/index.html). This study did not require ethics approval as all data were downloaded from public databases.

### Signature development and validation

Univariate Cox regression analysis was used to select RFS-related genes from the candidate gene list using the “survival” package in R software. Then, LASSO Cox regression analysis was performed to select optimal genes using the “glment”, “survminer”, and “survival” R packages. Thus, a prognostic ARG signature that calculates individual risk scores was developed based on the nonzero coefficients in the LASSO regression model. An autophagy-related signature for RFS was conducted based on expression levels for these genes and their corresponding coefficients (β). The risk score = (βmRNA1 × expression level of mRNA1) + (βmRNA2 × expression level of mRNA2) + (βmRNA3 × expression level of mRNA3) + (βmRNAn × expression level of mRNAn). BC patients were dichotomized into high- and low-risk groups based on the median value of the risk score. Survival outcomes of high- and low-risk score groups were then examined using a Kaplan-Meier survival plot. ROC curves were used to evaluate the performance of the FRG signature. The GSE22219 cohort was used as validation set to examine the versatility and reliability of the signature in a similar way. To validate the signature genes at the protein level, immunohistochemistry (IHC) images of both normal and BC samples were downloaded from the Human Protein Atlas database (https://www.proteinatlas.org/).

### Correlation of the ARG signature with clinicopathological characteristics

To explore the impact of the signature on the clinicopathological features of BC, we evaluated the correlation of the risk score with four clinicopathological factors (age, grade, and T and N stage).

### Nomogram construction

Univariate and multivariate Cox regression analyses were performed to evaluate whether the risk scores are independent prognostic factors for OS. The variables examined included age, ER and PR status (negative and positive), tumor grade, T stage, and N stage. Using *p* < 0.05 as the cut-off value, we performed univariate Cox’s proportional hazards regression analysis for these variables. Based on the backward stepwise method, we applied a multivariate Cox’s proportional hazards regression model to identify key variables. Then, a nomogram was constructed to predict 3- and 5-year RFS rates in BC patients. The nomogram thus obtained was validated by measuring its discrimination and calibration abilities. We used the area under the curve (AUC) to evaluate our model’s discrimination performance and to assess heterogeneities in its predicting ability (i.e. predicted vs observed results) using the “rms” R package.

### Statistical analyses

Continuous variables were expressed as mean ± standard deviation (SD) as appropriate. Chi-squared and *t*-tests were used to compare differences between two groups. The accuracy of the prognostic nomogram was assessed by the AUC values of ROC curves using the package “survivalROC” in R. Statistical analyses were performed with R software (version 3.6.1, http://www.r-project.org/).

### Availability of data and material

The GSE25066 and GSE22219 datasets analyzed in the current study are available from the GEO database (https://www.ncbi.nlm.nih.gov/geo/). Additional information about results of this work is available from the corresponding author upon reasonable request.
